# Supplementary material for: Ribociclib Induces Broad Chemotherapy Resistance and EGFR Dependency in ESR1 Wildtype and Mutant Breast Cancer
Source: Cancers (Basel). 2021 Dec 16;13(24):6314. doi: 10.3390/cancers13246314 (PMC8699146; doi:10.3390/cancers13246314)
Supplement: Supplementary file 1 [file cancers-13-06314-s001.zip › Supplementary Figures .pdf]

cancers

MDPI

Supplementary Materials: Ribociclib induces broad chemotherapy resistance and EGFR dependency in ESR1 wildtype and mutant breast cancer

Isabel Mayayo-Peralta, Beatrice Faggion, Liesbeth Hoekman, Ben Morris, Cor Lieftink, Isabella Goldsbrough, Lakjaya Buluwela, Joseph C Siefert, Harm Post, Maarten Altelaar, Roderick Beijersbergen, Simak Ali, Wilbert Zwart, and Stefan Prekovic

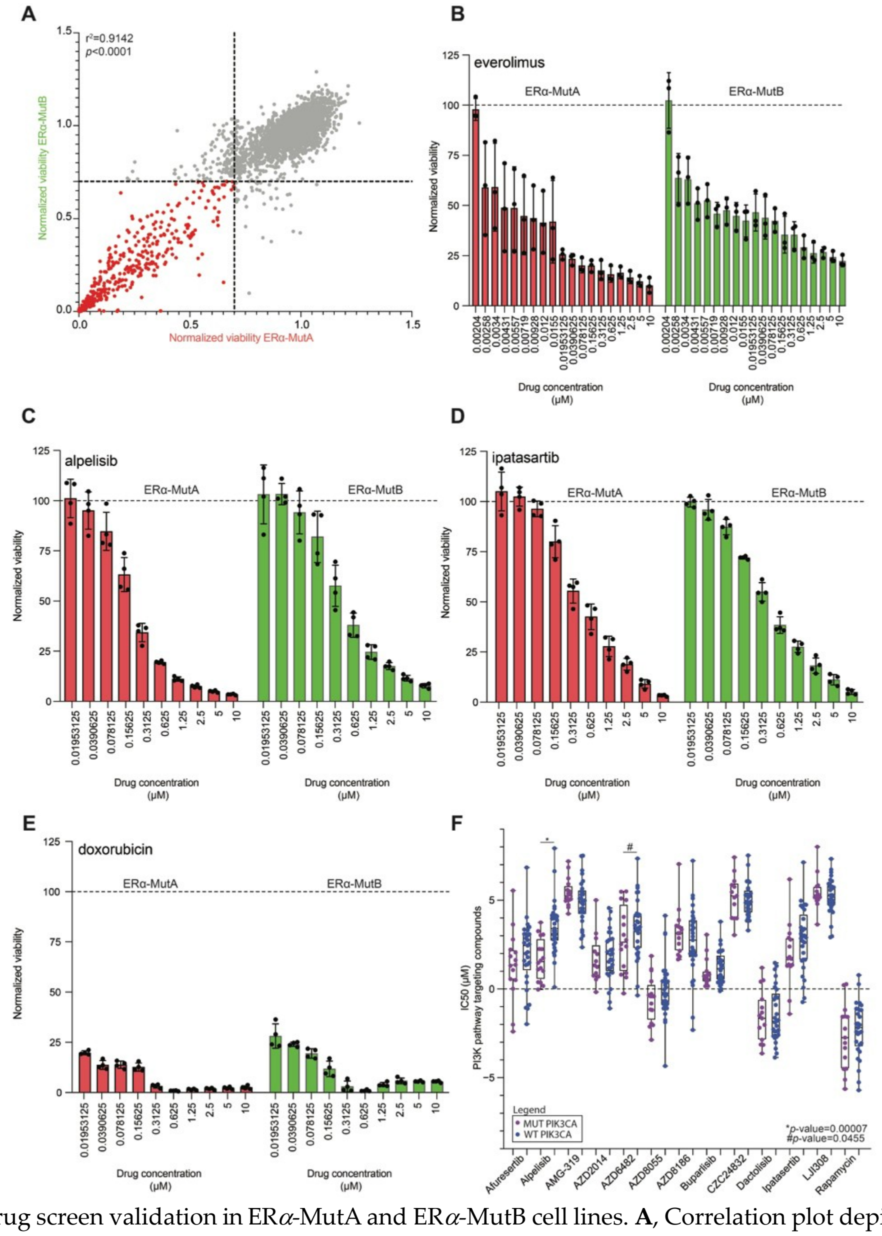

Figure S1. Drug screen validation in ERα-MutA and ERα-MutB cell lines. **A**, Correlation plot depicting normalized viability in both ERα-MutA and ERα-MutB cell lines for all compounds of the screen. Pearson r^2 and two-sided p-value is reported. **B-E**, ERα-MutA and ERα-WT cells were cultured with indicated inhibitor. After six days cell viability was measured using CellTiter-Glo. Bars depict mean value ±SD (everolimus n=3; alpelisib n=4; ipatasartib n=4, and doxorubicin =4). **F**, Sensitivity (reported IC50 values) of various breast cancer cell lines that harbour either *PI3KCA* wildtype (blue) or mutated (purple) allele to drugs targeting the PI3K pathway. The central mark of the boxplot indicates the median, and the bottom and top whiskers indicate the minimum and maximum, respectively. The Mann–Whitney U test was performed and a two-sided p-value is reported.

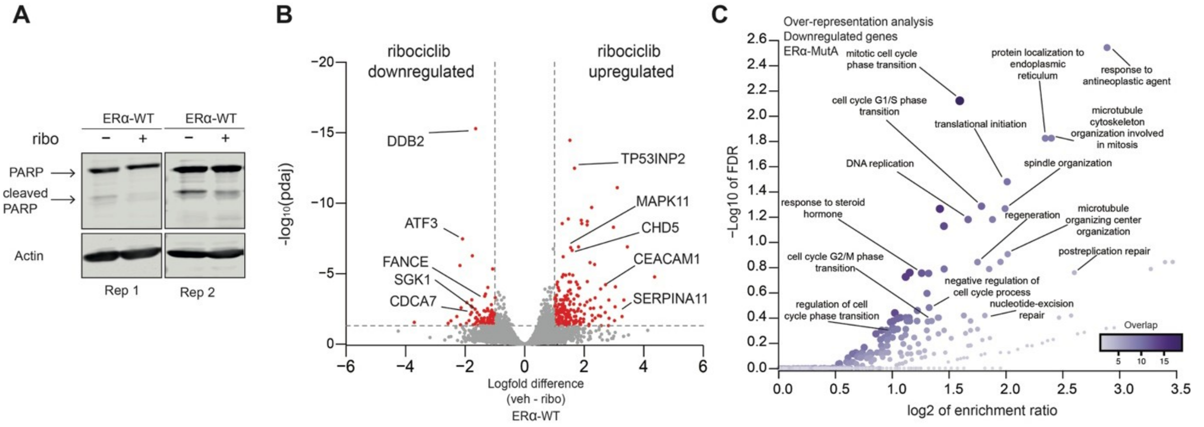

Figure S2. Ribociclib induces senescence in ERα-WT models. **A**, Representative western blot showing (cleaved) PARP without (-) and with ribociclib (+), with Actin as loading control (n=3). **B**, Volcano plot depicting log2fold differences from an RNA sequencing experiment of ERα-WT cells treated with either Vehicle or 600 nM ribociclib for five days (n=2). Differentially expressed genes (log2fold <-1 and >1; p-adj=0.05) are shown in red. Adjusted *p*-values (padj) were determined by DESeq2 (Wald test *p*-values were corrected for multiple testing using Benjamini and Hochberg method). **C**, Over-representation analysis of genes downregulated by ribociclib treatment in ERα-MutA cells (n=2). FDR was computed using the WebGestalt tool.

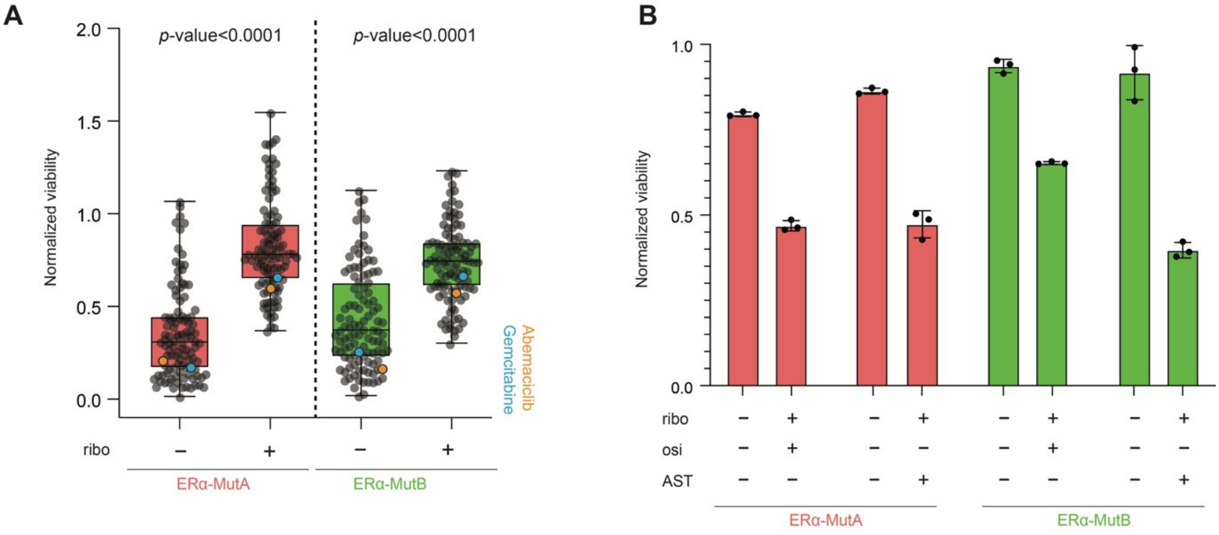

Figure S3. Ribociclib reduces effectiveness of various drugs and induces vulnerability to EGFR inhibitors. **A**, Normalized viability of ERα-MutA and ERα-MutB cell lines pre-treated (+) or not (-) with ribociclib (Ribo) and subsequently treated with compound libraries. Compounds displayed were selected based on their effect on ERα-MutA. Abemaciclib and gemcitabine are depicted in orange and blue, respectively. The central mark of the boxplot indicates the median, and the bottom and top edges of the box indicate the 25th and 75th percentiles, respectively. The maximum whisker lengths are specified as 1.5 times the interquartile range and outliers are depicted as empty circles. **B**, Normalized viability of ERα-MutA and ERα-MutB cell lines treated with ribociclib (ribo), osimertinib (osi), or AST-1306 (AST), or the combination. Bars represent mean values ±SD (n=3).

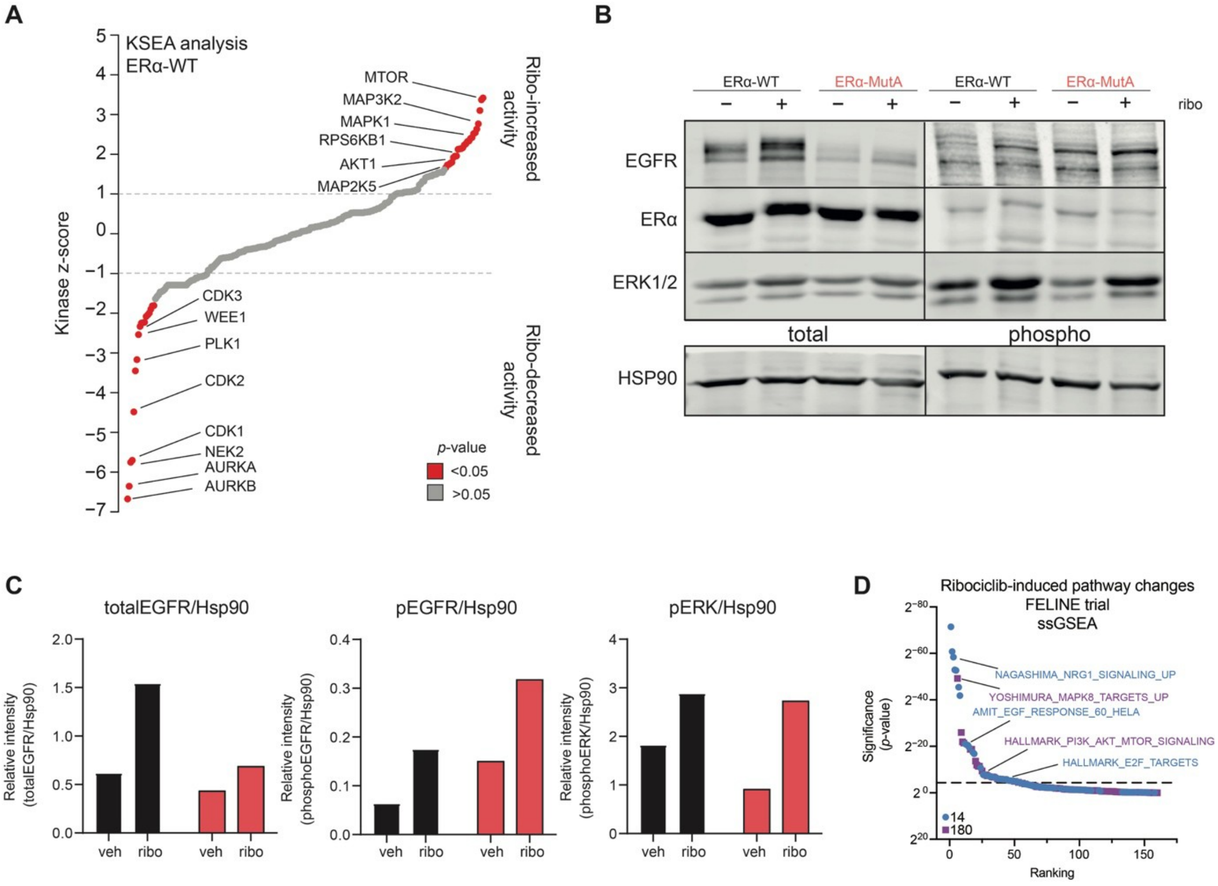

Figure S4. Ribociclib induces EGFR pathway activation. **A**, Plot depicting kinases ranked based on the kinase activity *z*-score calculated using kinase-substrate-enrichment-analysis of phospho-proteomic data (n=4). The significant kinases are mark red (*p*-value<0.05, determined by KSEA software). **B**, Representative western blot showing expression of both total and phosphorylated EGFR, ER*α*, and ERK1/2 in ER*α*-MutA and ER*α*-WT cell lines treated with ribociclib (+) or vehicle (-). Hsp90 was used as loading control (n=3). **C**, Quantification of the western blot from panel B. ImageJ was used to quantity the band intensity. **D,** Enrichment analysis for pathways related to cell cycle and EGFR/PI3K signaling specifically in breast cancer samples of patients treated with ribociclib. Statistical significance is based on hierarchical regression model of ssGSEA signatures. Two-sided *p*-values were corrected for multiple testing using Holm–Bonferroni method. Blue circles and purple squares represent comparison of baseline to 14- and 180-day treatment, respectively. Dotted line demarks *p*- value=0.05.

Figure1 panel C

ERα-WT

24hrs Fulvestrant

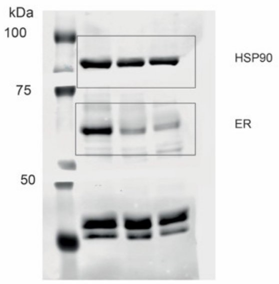

Figure1 panel C

ERα-MutA

24hrs Fulvestrant

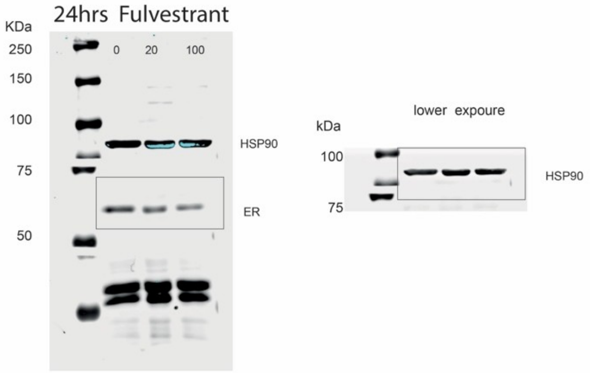

48hrs AZD

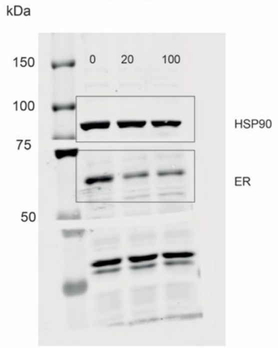

48hrs AZD

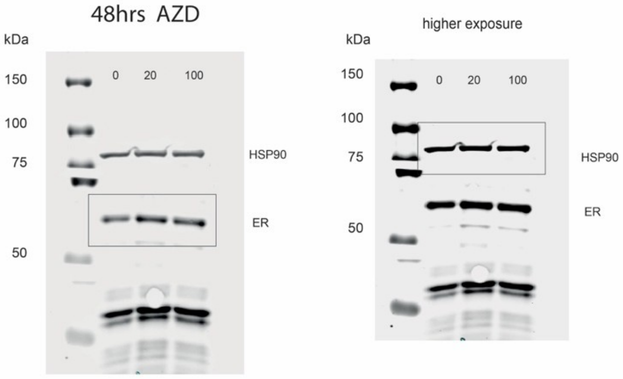

Supplementary Figure 2

Rep1

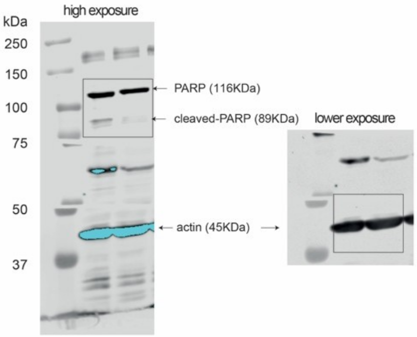

Rep2

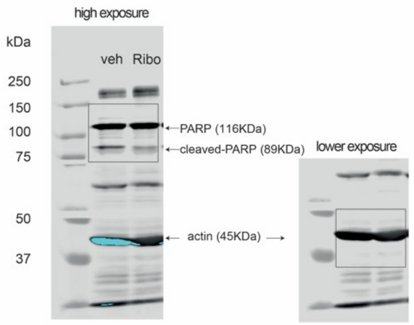

Supplementary Figure 4 panel b

From left to right, different exposures

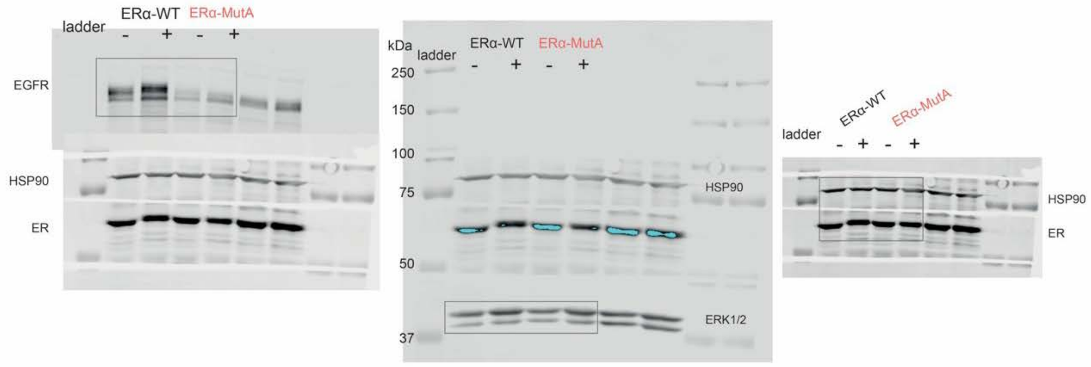

From left to right, different exposures

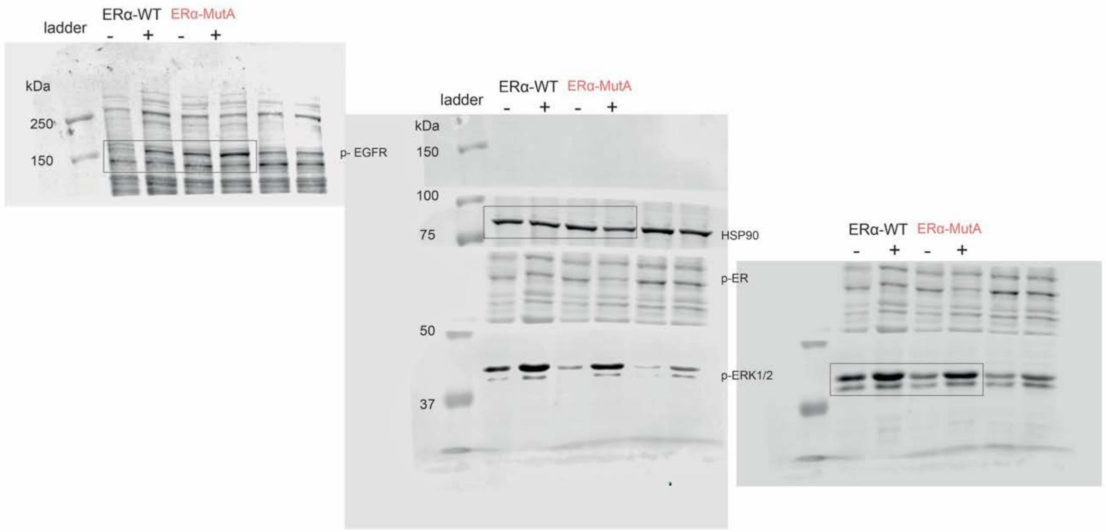

Figure S5. Uncropped western blot images.

MCF-7-ER mutA						
		ER	HSP90	ER/HSP90	ER/HSP90 (normalized to vehicle)	Figure 1 panel C
VHCL		9,7060	16,9420	0,5729	1	
ICI 20nM		5,8720	17,0750	0,3439	0,600274265	
ICI 100nM		4,3730	16,7180	0,2616	0,4565828	
		ER	HSP90	ER/HSP90	ER/HSP90 (normalized to vehicle)	
VHCL		24,2520	12,5930	1,9258	1	
AZD 20nM		39,9900	14,7320	2,7145	1,409520309	
AZD 100nM		35,7580	15,3740	2,3259	1,207724808	
MCF-7- WT						Figure 1 panel C
		ER	HSP90	ER/HSP90	ER/HSP90 (normalized to vehicle)	
VHCL		26,4770	39,8440	0,6645	1	
ICI 20nM		6,9720	31,1190	0,2240	0,337152125	
ICI 100nM		4,6530	28,9380	0,1608	0,241968424	
VHCL		20,8950	7,9540	2,6270	1	
AZD 20nM		7,9700	7,1250	1,1186	0,425810792	
AZD 100nM		6,3840	6,4710	0,9866	0,37554734	
PARP blots						Supplementary Figure 2
REP1		total PARP	Cleaved PARP	Actin	totalPARP/actin	
	VHCL	34,918	82,8620	12,1250	2,879835052	
	RIBO	32,5410	17,1380	11,6620	2,790344709	
					cleavedPARP/actin	
	VHCL				6,833979381	
	RIBO				1,469559252	
REP2		total PARP	Cleaved PARP	Actin	totalPARP/actin	Supplementary Figure 2
	VHCL	50,6100	62,4900	27,5420	1,837557185	
	RIBO	49,3900	37,5100	26,6970	1,850020602	
					cleavedPARP/actin	
	VHCL				2,26889841	
	RIBO				1,405026782	
EGFR blots						Supplementary Figure 4
		total EGFR	HSP90	EGFR/HSP90	EGFR/HSP90 (normalized to vehicle)	
MCF-7-WT	VHCL	4,2870	7	0,617812365	1	
	RIBO	9,627	6	1,541800128	2,495579913	
MCF-7-MutA	VHCL	2,829	7	0,431974347	1	
	RIBO	3,520	5	0,686427457	1,589046807	
phospho EGFR						Supplementary Figure 4
		p-EGFR	HSP90	p-EGFR/HSP90	p-EGFR/HSP90 (normalized to vehicle)	
MCF-7-WT	VHCL	0,398	6	0,063588433	1	
	RIBO	0,947	5	0,174691016	2,747213748	
MCF-7-MutA	VHCL	0,935	6	0,149935856	1	
	RIBO	1	4	0,317564509	2,118002435	
phospho ERK						Supplementary Figure 4
		p-ERK	HSP90	p-ERK/HSP90	p-ERK/HSP90 (normalized to vehicle)	
MCF-7-WT	VHCL	11	6	1,822655376	1	
	RIBO	16	5	2,880095923	1,58016483	
MCF-7-MutA	VHCL	6	6	0,908274535	1	
	RIBO	12	4	2,72589407	3,00117857	

Figure S6. Densitometry readings/intensity ratio of Western blot bands found across the figures of the manuscript.
